# Prevalence of hepatitis D virus infection among patients with chronic hepatitis B infection in a tertiary care centre in Thailand

**DOI:** 10.1038/s41598-023-49819-2

**Published:** 2023-12-19

**Authors:** Prooksa Ananchuensook, Sirinporn Suksawatamnuay, Panarat Thaimai, Nipaporn Siripon, Supachaya Sriphoosanaphan, Kessarin Thanapirom, Yong Poovorawan, Piyawat Komolmit

**Affiliations:** 1 Division of Gastroenterology, Department of Medicine, Faculty of Medicine, Chulalongkorn University, Rama4 Road, Patumwan, Bangkok, 10330 Thailand; 2 Center of Excellence in Liver Diseases, King Chulalongkorn Memorial Hospital, Thai Red Cross Society, Bangkok, Thailand; 3 Center of Excellence in Hepatic Fibrosis and Cirrhosis, Faculty of Medicine, Chulalongkorn University, Bangkok, Thailand; 4 Academic Affair, Faculty of Medicine, Chulalongkorn University, Bangkok, Thailand; 5 Center of Excellence in Clinical Virology, Faculty of Medicine, Chulalongkorn University, Bangkok, Thailand

**Keywords:** Hepatitis B, Epidemiology

## Abstract

Knowledge about the epidemiology of hepatitis D virus (HDV) is essential for effective screening and management. Our study aimed to update the prevalence of HDV infection among patients with hepatitis B virus (HBV) infection at hepatology clinics in Thailand. We enrolled HBV-infected patients from hepatology clinics at King Chulalongkorn Memorial Hospital, Bangkok, Thailand, between June 2022 and November 2023. Demographic, biochemical characteristics, and liver-related complications (LRC), including cirrhosis and hepatocellular carcinoma, were reviewed. The competitive enzyme and chemiluminescence immunoassays were used to detect anti-HDV antibodies. Real-time polymerase chain reaction (RT-PCR) was used to test for HDV RNA in anti-HDV-positive patients. The HDV genotype was identified in detectable HDV RNA samples. Of the 702 enrolled patients, four (0.6%) had positive and equivocal for both anti-HDV tests. Two (50.0%) of the four patients tested positive for HDV RNA and genotype 1 was identified; one had multiple risk factors. Anti-HDV seroprevalence was not significantly different between patients with and without LRC. In conclusion, HDV co-infection is less common in Thailand than globally. Additionally, our study identified genotype 1, the predominant HDV genotype worldwide, and observed co-infection even without LRC.

## Introduction

Chronic hepatitis D virus (HDV) and hepatitis B virus (HBV) co-infections lead to significantly higher risk (2.6 and 3.8-fold, respectively) of liver-related complications (LRC) and hepatocellular carcinoma (HCC)^1^. Previously, pegylated interferon-α was the only medication used to treat HDV. Recently, bulevirtide, an HDV entry inhibitor, was approved for HDV treatment in Europe^2,3^. Other emerging therapies, including lonarfanib, a prenylation inhibitor, and nucleic acid polymers, have led to an interest in the epidemiology and treatment of HDV infection^3–5^.

Recently, the prevalence of HBV infection was found to be lower after the introduction of the vaccination program. However, according to three recent meta-analyses by Chen et al., Miao et al., and Stockdale et al., the prevalence of hepatitis D infection in patients with hepatitis B was 14.6%, 13.0%, and 16.4%, respectively, which were higher than expected^6–9^. The epidemiology of HDV in Thailand was first reported 20 and 40 years ago^10–13^. In Thailand, the rate of anti-HDV positivity is higher in intravenous drug users (IVDU) (20–65%) than in non-IVDU with HBV infection (0–2%)^10–13^. Currently, patients with HBV infection are not routinely screened for HDV co-infection in Thailand. Therefore, our study provides an update on the prevalence of hepatitis D in Thai patients with HBV infection in general hepatology clinics. Understanding the epidemiology of hepatitis D will lead to the development of effective screening programs and therapeutic management strategies.

## Method

### Participants

Patients with chronic hepatitis B infection, defined as the presence of persistent hepatitis B antigen (HBsAg) for at least six months, at the hepatology and hepatoma clinics at the King Chulalongkorn Memorial Hospital (KCMH), from June 2022 to October 2023 were enrolled in this study. KCMH is an academic tertiary hospital in Bangkok, Thailand. Currently, three distinct government health insurance schemes exist in Thailand, each with its unique features. Firstly, the Civil Servant Medical Beneficiary System (CMBS), organized by the Ministry of Finance, covers civil servants and their immediate family members. Individuals under CMBS are allowed to access any government hospital, including KCMH. Secondly, the Social Security Scheme (SSS), under the Ministry of Labor, is funded by triparty payments from employers, employees, and the government. SSS supports workforce labor in the private sector. Lastly, Universal Health Coverage (UHC), administered by the National Health Security Office, provides coverage to the rest of the Thai population. Individuals with SSS or UHC are required to seek medical care at their designated primary hospital. If the primary hospitals are unable to offer the necessary treatment, they transfer patients to secondary and tertiary hospitals, which include KCMH. Hence, individuals seeking care at KCMH may have their expenses covered by one of three government health insurance options, or they may be self-paying or covered by private health insurance. Information regarding patients' healthcare coverage and demographic region was obtained from electronic records within the KCMH administration system.

Baseline characteristics including age, sex, presence of HBV e antigen (HBeAg), HBV viral load (HBV VL), HBV treatment, and co-infection with hepatitis C virus (HCV) or human immunodeficiency virus (HIV) were collected. In addition, liver function test (LFT) results and alpha-fetoprotein (AFP) levels within six months before blood collection were recorded.

Data on liver-related outcomes, including cirrhosis, liver decompensation, and HCC, were collected from medical records at enrolment. Cirrhosis was diagnosed by radiologic findings according to the AASLD guideline. Liver decompensation included the presence of variceal bleeding and ascites. HCC was diagnosed by the presence of an arterial-enhancing liver mass on contrast-enhanced abdominal imaging or by histopathology. Barcelona stage and HCC treatment were also identified from the chart review.

**Figure 1:**
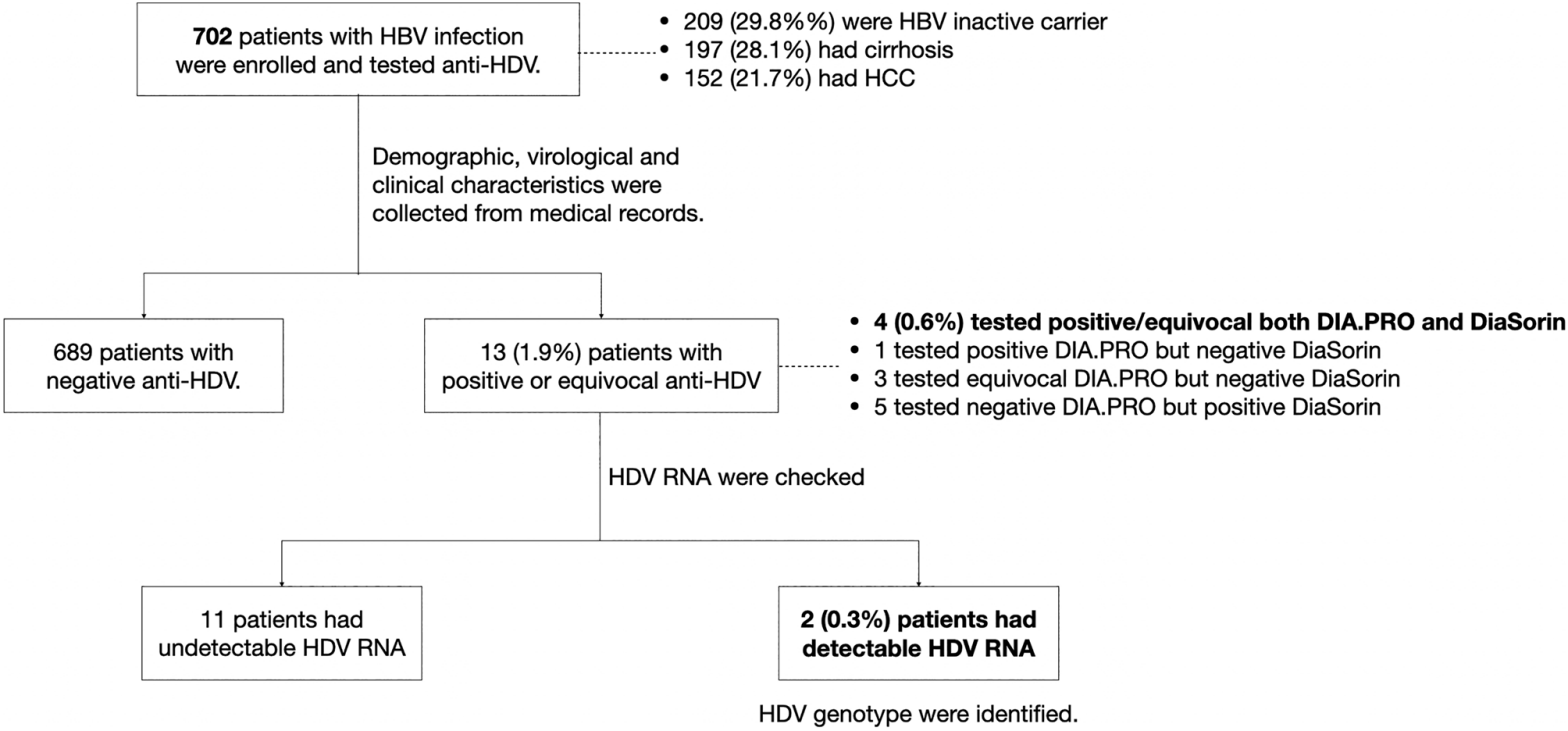
Flow chart of study design. *Anti-HDV* anti-hepatitis D virus antibody, *HBV* hepatitis B virus, *HCC* hepatocellular carcinoma, *HDV* hepatitis D virus, *RNA* ribonucleic acid.

First, all patients were tested for the presence of anti-hepatitis D virus antibodies (anti-HDV). Anti-HDV-positive patients were interviewed regarding risk factors of blood-borne infections, including IVDU, men who have sex with men (MSM), multiple partners, tattooing, and history of blood transfusion. Subsequently, HDV ribonucleic acid (RNA) and genotypes were analysed in anti-HDV-positive patients. A flowchart of the study design is shown in Fig. 1.

### Serological assay

All participants were tested for anti-HDV antibodies with both DIA.PRO and DiaSorin immunoassay. Plasma samples were tested for anti-HDV antibodies according to the manufacturer’s instructions using a competitive enzyme immunoassay HDV antibody (DIA.PRO, Italy). Anti-HDV results were interpreted based on the ratio between the cut-off value and the sample (Co/S). A Co/S value of 0.9–1.1 was considered equivocal, while a value of > 1.1 was considered positive for anti-HDV antibodies.

As stated by the manufacturer, DIA.PRO is reported to have a sensitivity and specificity exceeding 98%.

Simultaneously, serum samples were tested for the qualitative determination of antibodies to the hepatitis D virus (anti-HDV) using chemiluminescence immunoassay (CLIA) technology LIAISON® XL MUREX Anti-HDV (DiaSorin Biotechnology, Italy). The reaction was performed on LIAISON® XL. The cut-off value discriminating between the presence and the absence of HDV antibodies is 1.00 arbitrary unit per milliliter (AU/mL)., in which Specimens with AU/mL above or equal to 1.00 are considered reactive for HDV antibodies. As per the manufacturer's information, DiaSorin is reported to have a sensitivity of 99.5% and a specificity of 98.3–99.9%

### HDV-RNA detection

In patients positive for anti-HDV antibodies, HDV RNA was subsequently tested to diagnose the HDV infection. RNA was extracted from 200 μL of plasma using the Automatic Nucleic Acid Extraction System magLEAD (Precision System Science Co., Ltd., Chiba, Japan). The detection of HDV RNA was performed using real-time polymerase chain reaction (PCR) (RealStar® HDV RT-PCR Kit 1.0; Altona, Germany) according to the manufacturer’s instructions. The lower limit of detection of the real-time PCR assay was 1 × 10^–2^ international unit per microliter (IU/µL). The amplification process was performed using the LightCycler 480 Instrument II (Roche, Switzerland).

### HDV genotype sequencing

Positive HDV RNA samples were used to determine the HDV genotype by RT-PCR. Complementary DNA was synthesised from 10 μL of extracted RNA using a High-Capacity cDNA Reverse Transcription Kit (Applied Biosystems, Foster City, CA, USA) according to the manufacturer’s instructions. The partial HDV antigenomic ribozyme gene was amplified with the published primers 120, TH1, 118^10^ and R0^14^ PCR was performed under the following conditions: 5 µL of cDNA, 12.5 µL of AccuStart II GelTrack PCR Super Mix (QuantaBio, Beverly, MA), 0.25 µM forward and reverse primers, and a final adjustment to 25 µL total reaction volume. The PCR conditions were pre-incubation at 95 °C for 3 min, 40 cycles of denaturation at 95 °C for 45 s, annealing at 55 °C for 45 s, extension at 72 °C for 60 s, and final extension at 72 °C for 5 min. Amplicons of approximately 441 and 400 base pairs (bp) were purified and subjected to Sanger sequencing. Nucleotide sequences were analysed using BLASTN (http://www.ncbi.nlm.nih.gov) and the final genotypes were determined by phylogenetic analysis. Maximum likelihood trees were generated using the Hasegawa-Kishino-Yano + Gamma distributed model with 1000 bootstrap replicates in MEGA11 (http://www.megasoftware.net). The nucleotide sequences obtained were deposited in the GenBank database under the accession number OQ921609. The nucleotide sequences were compared with the reference strains using the neighbour-joining method with bootstrap consensus inferred from 1000 replicates. Bootstrap values > 70% are shown at the branch nodes. The scale bar represents the nucleotide substitution rate.

### Statistical analysis

For baseline characteristics, categorical variables are presented as frequency (%) and continuous variables as mean with standard deviation (SD) or median with interquartile range. Comparisons of variables in patients with anti-HDV-positive and anti-HDV-negative statuses were performed using the chi-square test or Fisher’s exact test for categorical data and using the t-test or Mann–Whitney *U* test for continuous variables. All statistical analyses were performed using SPSS software (version 22.0; IBM Corp., NY, USA).

This study was reviewed and approved by the Ethics Committee and Institutional Review Board of the Faculty of Medicine, Chulalongkorn University, Bangkok, Thailand (IRB Number: 268/65). The study protocol was performed in accordance with the Declaration of Helsinki (1989) of the World Medical Association.

### Ethics approval

Informed consent was obtained from participants. The study was reviewed and approved by the Ethics Committee and Institutional Review Board of the Faculty of Medicine, Chulalongkorn University, Bangkok, Thailand (IRB Number: 268/65).

## Results

### Baseline characteristics of enrolled patients

A total of 702 HBV-infected patients were enrolled from the hepatology and hepatoma clinics at KCMH. Of these, 418 patients (59.5%) were male, and the mean age was 54.01 ± 12.01 years. Regarding health coverage, most patients had CMBS (29.1%), followed by self-pay or private health insurance (28.5%). In addition, 372 (53.0%) of enrolled patients lived in Bangkok, followed by Central region of 240 (34.2%) (Supplementary Table 1).

Approximately 90% of the co-infection data were accessed; only one and four patients had HCV and HIV, respectively. For HBV infection, 452 patients (64.3%) were negative for HBeAg. Additionally, 209 (29.8%) were classified as HBV carriers, defined by patients with HBV VL below 2000 IU/mL, no evidence of significant liver fibrosis, and without indications for HBV treatment. Meanwhile, 471 (67.1%) have already initiated HBV antiviral treatment. In addition, 197 (28.1%) and 152 (21.7%) patients developed cirrhosis and HCC, respectively (Table 1).

**Table 1:** Characteristics of enrolled patients (N = 702).

Characteristics	Number (%)/mean ± SD
Male sex	418 (59.5%)
Age (in years)	54.01 ± 12.01
Virological characteristics	
HBeAg	Positive 89 (12.7%) Negative 452 (64.3%) Missing data 161 (22.9%)
Anti-HCV	Positive 4 (0.6%) Negative 637 (90.7%) Missing data 61 (8.7%)
Anti-HIV	Positive 1 (0.1%) Negative 635 (90.5%) Missing data 66 (9.4%)
Clinical characteristics	
HBV carriers	209 (29.8%)
Under HBV antiviral treatment	471 (67.1%)
Regimen, N = 471	
Lamivudine (LAM)	29 (6.2%)
Entecavir (ETV)	172 (36.5%)
Tenofovir (TDF & TAF)	258 (54.8%)
Combined ETV and TDF/TAF	11 (2.3%)
Interferon	1 (0.2%)
Cirrhosis (with and without HCC)	197 (28.1%)
CTP, N = 197	
A	166 (84.3%)
B	22 (11.2%)
C	9 (4.6%)
Hepatocellular carcinoma (HCC)	152 (21.7%)
Active	80 (11.4%)
BCLC	
A	23 (28.7%)
B	17 (21.3%)
C	40 (50.0%)
Ascites	24 (3.4%)
Variceal bleeding	22 (3.1%)

*Anti-HCV* Hepatitis C antibody, *anti-HIV* Human immunodeficiency virus antibody, *BCLC* Barcelona Clinic Liver Cancer, *CTP* Child–Turcotte–Pugh, *HBeAg* Hepatitis B e-antigen, *SD* standard deviation.

### Anti-HDV prevalence, HDV RNA and HDV genotype

Among the 702 samples from patients with HBV infection, four (0.6%) were positive, and four (0.6%) were equivocal for anti-HDV antibodies using DIA.PRO immunoassay. On the other hand, nine (1.3%) were positive for anti-HDV using DiaSorin immunoassay. The Anti-HDV levels of thirteen samples, which had positive or inconclusive results from either DIA.PRO or DiaSorin, were presented in Table 2. Subsequently, thirteen patients with positive and equivocal anti-HDV titres were tested for HDV RNA (Fig. 1).

**Table 2:** The anti-HDV titer, HDV RNA, and HDV genotype among patients with a positive or equivocal anti-HDV status determined by either DIA.PRO or DiaSorin assay.

No.	Anti-HDV				HDV RNA (Cp)	HDV RNA (IU/ml)	HDV genotype
	DIA.PRO	Titre (Co/S)	DiaSorin	Titre (AU/mL)			
1*	Positive	8.9	Positive	21.7	31.07	159.75	1
2*	Positive	9.0	Positive	23.2	33.19	35.5	–
3*	Positive	5.9	Positive	22.9	Undetectable	Undetectable	–
4	Positive	8.9	Negative	< 1.0	Undetectable	Undetectable	–
5*	Equivocal	1.1	Positive	13.9	Undetectable	Undetectable	–
6	Equivocal	1.0	Negative	< 1.0	Undetectable	Undetectable	–
7	Equivocal	0.99	Negative	< 1.0	Undetectable	Undetectable	–
8	Equivocal	0.92	Negative	< 1.0	Undetectable	Undetectable	–
9	Negative	0.32	Positive	2.98	Undetectable	Undetectable	–
10	Negative	0.34	Positive	2.87	Undetectable	Undetectable	–
11	Negative	0.63	Positive	1.98	Undetectable	Undetectable	–
12	Negative	0.48	Positive	1.02	Undetectable	Undetectable	–
13	Negative	0.44	Positive	1.02	Undetectable	Undetectable	–

*Anti-HDV positive group.

*anti-HDV* Hepatitis D antibody, *AU/mL* arbitrary unit per milliliter, *Co/S* Ratio between Cut-off value and the sample, *HDV* Hepatitis D virus, *IU/mL* international unit per millilitre, *No* number.

**Figure 2:**
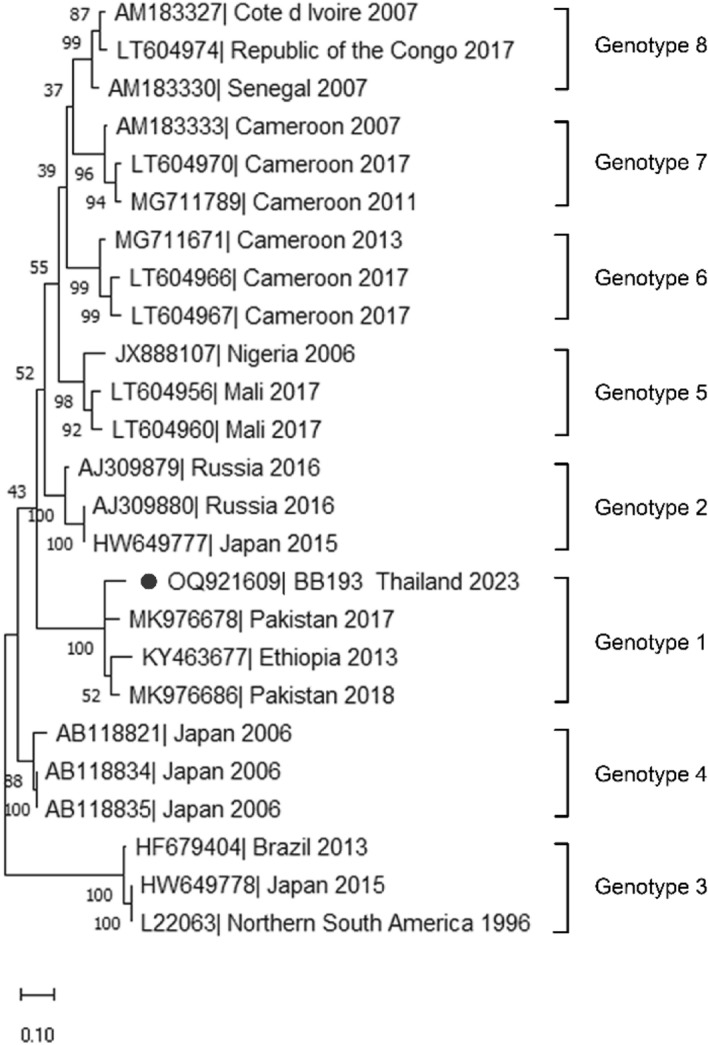
Phylogenetic tree based on the partial HDV antigenomic ribozyme sequences derived from the serum sample of one of the two patients with chronic hepatitis D. The black dot shown in the figure represents the study case.

HDV RNA was identified in two out of thirteen anti-HDV-positive and equivocal cases. Therefore, the prevalence of detectable HDV RNA in our study cohort was only 0.3%. The serum HDV RNA levels in both patients are shown in Table 2. One HDV-positive specimen with sufficient viral load was selected for genomic sequencing. Phylogenetic analysis was performed and genotype 1 was identified in the samples (Fig. 2).

### Details of anti-HDV-positive cases and comparison of characteristics between anti-HDV-positive and anti-HDV-negative patients

Four (0.6%) individuals who showed positive or inconclusive results on both DIA.PRO and DiaSorin tests were classified into the anti-HDV-positive group. Only one patient with positive anti-HDV and HDV RNA had multiple identified risk factors for HDV infection, including a history of IVDU, multiple sex partners, MSM, and HCV co-infection (Supplementary Table 3).

Comparisons of the demographic, virological, clinical, and LFT results between patients with negative and positive anti-HDV results are shown in Tables 3 and Supplementary Table 4. HCV co-infection was statistically different between groups, while other clinical outcomes and laboratory values, including alanine aminotransferase (ALT), were not statistically different between groups (Tables 3 and Supplementary Table 4). When focusing on clinical characteristics, all four patients in the positive anti-HDV group received antiviral therapy. Among them, one (25.0%) had decompensated cirrhosis and variceal bleeding, while none had HCC. Nevertheless, two patients with detectable HDV RNA did not have cirrhosis (Table 3 and Supplementary Table 3).

**Table 3:** Comparison of clinical and virological characteristics of anti-HDV positive and anti-HDV negative patients.

Characteristics	Anti-HDV negative n = 698	Anti-HDV positive n = 4	p-value
	Number (%)/mean ± SD	Number (%)/mean ± SD	
Male sex	415 (59.5%)	3 (75%)	0.651
Age (in years)	54.01 ± 12.04	52.50 ± 6.56	0.837
Virological characteristics			1.000
HBeAg	N = 537		
Positive	89 (16.6%)	0 (0%)	
Negative	448 (83.4%)	4 (100%)	
Positive anti-HCV	3/637 (0.5%)	1 (25.0%)	0.025*
Positive anti-HIV	1/632 (0.2%)	0 (0%)	1.000
Clinical characteristics			
HBV carriers	209/698 (29.9%)	0/4 (0%)	0.324
Cirrhosis	196 (28.1%)	1 (25.0%)	1.000
Hepatocellular carcinoma	152 (21.8%)	0 (0%)	0.574
Ascites	24 (3.4%)	0 (0%)	1.000
Variceal bleeding	21 (3.0%)	1 (25.0%)	0.120

*p-value < 0.05, Fischer’s Exact test.

*AFP* alpha-fetoprotein, *anti-HCV* Hepatitis C antibody, *anti-HDV* Hepatitis D antibody, *anti-HIV* Human immunodeficiency virus antibody, *BCLC* Barcelona Clinic Liver Cancer, *HBeAg* Hepatitis B e-antigen.

## Discussion

HDV/HBV co-infection leads to adverse clinical outcomes, including liver cirrhosis, liver failure, and HCC within a decade after dual infection^15^. However, the routine screening and monitoring of HDV co-infection in patients with chronic hepatitis B infection is not widely implemented in clinical practice^16,17^. In Thailand, vertical transmission is the primary route of HBV transmission^18^. Although the introduction of universal HBV vaccination in 1988 has led to a reduction in new HBV infections, HBsAg seropositivity among individuals aged 35–64 years remains at 6%^19,20^. Considering that Thailand is an endemic area for HBV infection, an updated assessment of the prevalence of HDV co-infection in Thailand is crucial. Our cohort reported a 0.6% prevalence of HDV seropositivity and 0.3% of HDV current infection determined by detectable HDV RNA.

Three recent meta-analyses revealed that the global prevalence of HDV infection among HBsAg-positive patients in hepatology clinics is approximately 13–16.5%^8,9^. The estimated anti-HDV prevalence was lower (4.0%) in pooled data from Southeast Asia region, including Indonesia, India, Bangladesh, and Thailand^8^. Therefore, our study confirmed that the seroprevalence of HDV in Thailand remains lower compared to both the global and regional prevalence rates. We hypothesized that the lower prevalence of HDV co-infection in HBV-infected patients in Thailand resulted from HBV transmission route primarily from mothers to children and a successful HIV prevention campaign that reduced risks, such as unsafe sex and IVDU, both of which are associated with HDV infection. Nevertheless, the impact of ethnic and geographical factors on HDV infection rates requires exploration^18,21^.

Supplementary Table 5 provides a summary of the anti-HDV seroprevalence reports in Thailand. As outlined in the table, the prevalence of anti-HDV varies depending on the population studied. The high positive HDV seroprevalence of 65.48% and 21.8% was observed in IVDU with HBV infection in Thailand, while it remained undetected in all asymptomatic blood donors with positive HBsAg^10,12,13^. Furthermore, The latest two studies on minorities in northern Thailand showed a low prevalence of HDV co-infection at 0 and 0.7%^22,23^. In hospital settings, Chainuvathi et al. and Louisirirotchanakul et al. demonstrated HDV seroprevalence among HBV-infected patients in Thailand of 0.5% and 2.22%, respectively^10,12^. Notably, both studies observed a higher prevalence of approximately 8% among patients with hepatitis and cirrhosis^11,12^. Our study updated the HDV seroprevalence in a tertiary care setting in Thailand, revealing a similar low anti-HDV prevalence of 0.6% compared to previous hospital-based studies in Thailand. Although one patient had multiple risk factors for HDV infection, the remaining three patients did not exhibit any identifiable risk factors. The strength of our study is the large number of patients in the total cohort and the inclusion of more than 150 patients with HCC. These findings highlight that HDV co-infection persists, even in regions with lower HBV infection rates.

In addition to determining HDV seroprevalence, we also revealed a prevalence of 0.3% for the detected HDV RNA and identified HDV genotype 1 in our cohort. Although HDV RNA is replicating, quantitative measurements are not routinely performed^24^. The detection rate of HDV RNA in previous studies varied from 30 to 70% in patients who tested positive for anti-HDV antibodies^1,25^. Our study is the second study in Thailand that reported detectable HDV RNA prevalence and we found a proportion of 50.0% which is comparable to previous reports. Genotype 1 is the most common genotype globally, whereas genotypes 2 and 4 are localised in Asia^8,24^. Only one study has reported HDV genotype 1 in IVDU infected with HBV in Thailand^10^. We identified HDV genotype 1 in our cohort, and our findings were consistent with those of a previous study that HDV genotype 1 is the most prevalent genotype worldwide.

Our study did not find statistically significant differences in the anti-HDV prevalence between patients with cirrhosis, HCC, and those with asymptomatic HBV infection. This can be attributed to the low overall prevalence of HDV infection in our cohort. A long-term follow-up of these participants is necessary to determine the development of LRC in patients with HDV co-infection.

Our study provides an update on the prevalence of anti-HDV positivity among patients with HBV infection in a hepatology clinic in Thailand. However, our study has some limitations. Firstly, the cross-sectional design of the study limits our ability to assess the clinical course and long-term outcomes of patients with concomitant HDV and HBV infections. To gain a more comprehensive understanding of the clinical implication and prognosis of co-infection, a longitudinal study would be necessary. Secondly, the assessment of HDV infection risk was based on self-reported interviews, which may introduce the possibility of recall bias. While we made efforts to ensure the accuracy of the information provided by the participants, there remains the potential for incomplete verification of risk factors. Lastly, around 50% of the participants in our study resided in metropolitan areas. This can be attributed to the healthcare referral system in Thailand, which applies to individuals covered by UHC and SSS. These individuals generally initiate their medical treatment at their local primary hospital and are not obligated to transfer to a tertiary care facility, such as KCMH, for HBV treatment.

The epidemiology of hepatitis D in hepatology clinics in Thailand provides information for designing effective screening programs and therapeutic management strategies for hepatitis D in Thailand and other non-endemic countries. Despite the relatively low prevalence of HDV infection, our study identified a group of patients who did not exhibit high-risk factors or develop LRC. With the increasing availability of HDV treatment, these patients could potentially benefit from HDV eradication to halt the progression of their chronic liver disease. Nevertheless, further cost-effectiveness studies are required to recommend HDV infection surveillance for all patients with HBV infection, particularly in low-prevalence countries.

### Supplementary Information

Supplementary Tables.

## Data Availability

The HDV RNA nucleotide sequences obtained were deposited in the GenBank database under the accession number OQ921609. Other data that support the findings of this study are available from the corresponding author upon reasonable request.

## References

[1] Kamal H, Westman G, Falconer K, Duberg AS, Weiland O, Haverinen S (2020). Long-term study of hepatitis delta virus infection at secondary care centers: The impact of viremia on liver-related outcomes. Hepatology..

[2] Koh C, Heller T, Glenn JS (2019). Pathogenesis of and new therapies for hepatitis D. Gastroenterology..

[3] Urban S, Neumann-Haefelin C, Lampertico P (2021). Hepatitis D virus in 2021: Virology, immunology and new treatment approaches for a difficult-to-treat disease. Gut..

[4] Khan IW, Dad Ullah MU, Choudhry M, Ali MJ, Ali MA, Lam SLK (2021). Novel therapies of hepatitis B and D. Microorganisms..

[5] Yardeni D, Heller T, Koh C (2022). Chronic hepatitis D: What is changing?. J. Viral Hepat..

[6] Papatheodoridi M, Papatheodoridis GV (2021). Current status of hepatitis delta. Curr. Opin. Pharmacol..

[7] Chen HY, Shen DT, Ji DZ, Han PC, Zhang WM, Ma JF (2019). Prevalence and burden of hepatitis D virus infection in the global population: A systematic review and meta-analysis. Gut..

[8] Stockdale AJ, Kreuels B, Henrion MYR, Giorgi E, Kyomuhangi I, de Martel C (2020). The global prevalence of hepatitis D virus infection: Systematic review and meta-analysis. J. Hepatol..

[9] Miao Z, Zhang S, Ou X, Li S, Ma Z, Wang W (2020). Estimating the global prevalence, disease progression, and clinical outcome of hepatitis delta virus infection. J. Infect. Dis..

[10] Theamboonlers A, Hansurabhanon T, Verachai V, Chongsrisawat V, Poovorawan Y (2002). Hepatitis D virus infection in Thailand: HDV genotyping by RT-PCR RFLP and direct sequencing. Infection.

[11] Chainuvati T (1987). The prevalence of delta infection among Thai hepatitis B carriers. Intern. Med..

[12] Louisirirotchanakul S, Wasi C, Uneklabh C, Phutiprawan T, Suwanagool S, Chainuvati T (1988). High prevalence of delta virus infection in Thai intravenous drug abusers. Southeast Asian J. Trop. Med. Public Health..

[13] Jutavijittum P, Jiviriyawat Y, Yousukh A, Kunachiwa W, Hayashi S, Toriyama KJ (2002). Short communication seroprevalence of hepatitis D virus infection among HBsAg carriers in northern Thailand. Jpn. J. Trop. Med. Hyg..

[14] Le Gal F, Gault E, Ripault MP, Serpaggi J, Trinchet JC, Gordien E (2006). Eighth major clade for hepatitis delta virus. Emerg. Infect. Dis..

[15] Khalfi P, Kennedy PT, Majzoub K, Asselah T (2023). Hepatitis D virus: Improving virological knowledge to develop new treatments. Antiviral Res..

[16] Terrault NA, Lok AS, McMahon BJ, Chang KM, Hwang JP, Jonas MM (2018). Update on prevention, diagnosis, and treatment of chronic hepatitis B: AASLD 2018 hepatitis B guidance. Hepatology.

[17] Sulkowski MS (2023). Hepatitis D virus infection: Progress on the path toward disease control and cure. J. Viral Hepat..

[18] Poovorawan Y, Chongsrisawat V, Tangkijvanich P (2001). Problems and prevention of viral hepatitis in Thailand. J. Med. Assoc. Thailand..

[19] Posuwan N, Wanlapakorn N, Sa-Nguanmoo P, Wasitthankasem R, Vichaiwattana P, Klinfueng S (2016). The success of a universal hepatitis B immunization program as part of Thailand's EPI after 22 years' implementation. PLoS ONE..

[20] Posuwan N, Wanlapakorn N, Sintusek P, Wasitthankasem R, Poovorawan K, Vongpunsawad S (2020). Towards the elimination of viral hepatitis in Thailand by the year 2030. J. Virus Erad..

[21] Harris J, Thaiprayoon S (2022). Common factors in HIV/AIDS prevention success: Lessons from Thailand. BMC Health Serv. Res..

[22] Hongjaisee S, Khamduang W, Sripan P, Choyrum S, Thepbundit V, Ngo-Giang-Huong N (2020). Prevalence and factors associated with hepatitis B and D virus infections among migrant sex workers in Chiangmai, Thailand: A cross-sectional study in 2019. Int. J. Infect. Dis..

[23] Louisirirotchanakul S, Myint KS, Srimee B, Kanoksinsombat C, Khamboonruang C, Kunstadter P (2002). The prevalence of viral hepatitis among the Hmong people of northern Thailand. Southeast Asian J. Trop. Med. Public Health..

[24] Usai C, Gill US, Riddell AC, Asselah T, Kennedy PT (2022). Review article: Emerging insights into the immunopathology, clinical and therapeutic aspects of hepatitis delta virus. Aliment Pharmacol. Ther..

[25] Servant-Delmas A, Le Gal F, Gallian P, Gordien E, Laperche S (2014). Increasing prevalence of HDV/HBV infection over 15 years in France. J. Clin. Virol..

