# Supplementary material for: Prevalence of hepatitis D virus infection among patients with chronic hepatitis B infection in a tertiary care centre in Thailand
Source: Sci Rep. 2023 Dec 19;13:22633. doi: 10.1038/s41598-023-49819-2 (PMC10730816; doi:10.1038/s41598-023-49819-2)
Supplement: Supplementary file 1 — Supplementary Tables. [file 41598_2023_49819_MOESM1_ESM.docx]

Supplement table 1 Health coverage and demographic data of the entire cohort

| **Health coverage and demographic data** | **Number (%)** |
| --- | --- |
| Health coverage  UHC  CMBS  SSS  Self-pay or private health insurance | 185 (26.3%)  204 (29.1%)  113 (16.1%)  200 (28.5%) |
| Demographic data  Metropolitan region (Bangkok)  Rural regions   - Northern - Northeastern - Central - Southern | 372 (53.0%)  330 (47.0%)   - 40 (12.1 %) - 38 (11.5%) - 240 (72.7%) - 12 (3.7%) |

CH, chronic hepatitis; CMBS, Civil Servant Medical beneficiary System; HCC, hepatocellular carcinoma; IC, inactive carrier; SSS, Social Security Scheme; UHC, Universal Health Coverage.

Supplement table 2 Liver function tests and alpha-fetoprotein of the entire cohort

| **Laboratory findings** | **Number (%)/ Median (IQR)** |
| --- | --- |
| Albumin (g/dL), N = 682  TB (mg/dL), N = 686  AST (U/L, N = 694  ALT (U/L), N = 694  Platelet (x10^3^/µL), N = 673  AFP (ng/mL), N = 646 | 4.30 (4.10-4.50)  0.68 (0.51-0.98)  24.00 (20.00-34.00)  24.00 (18.00-36.00)  217.00 (173.0-261.50)  2.20 (1.51-2.60) |

AFP, alpha-fetoprotein; ALT, alanine aminotransferase; AST, aspartate aminotransferase; g/dL, gram/deciliter; mg/dL, milligram per deciliter; ng/mL, nanogram per milliliter; SD, standard deviation; TB, total bilirubin; U/L, units per liter; µL, microliter

Supplement table 3 Characteristics, risk factors of blood-borne infection, co-infection, details of HBV infection, and liver related complications of patients with positive and equivocal anti-HDV.

| **No.** | **Sex** | **Age**  **(years)** | **Risk factors for blood-borne infection** | | | | | **Anti-HIV** | **Anti-HCV** |
| --- | --- | --- | --- | --- | --- | --- | --- | --- | --- |
|  |  |  | **IVDU** | **multiple partners** | **tattooing** | **Blood transfusion** | **MSM** |  |  |
| 1 | Male | 43 | Yes | Yes | No | No | Yes | Negative | Positive |
| 2 | Male | 55 | No | No | No | No | No | Negative | Negative |
| 3 | Male | 54 | No | No | No | No | No | Negative | Negative |
| 5 | Female | 58 | No | No | No | No | - | Negative | Negative |

| **No.** | **HBV infection** | | | | | **ALT (U/L)** | **LRC** | |
| --- | --- | --- | --- | --- | --- | --- | --- | --- |
|  | **FH of HBV** | **HBeAg** | **HBV VL (IU/mL)** | **Under antiviral Rx/ regimen** | **Duration of HBV Rx** |  | **Cirrhosis** | **HCC** |
| 1 | No | Negative | < 10 | Yes/ETV | 1yr, 4mo | 89.0 | No | No |
| 2 | No | Negative | < 10 | Yes/LAM & ADV | 15yr, 5mo | 48.0 | No | No |
| 3 | No | Negative | < 10 | Yes/ ETV | 1yr, 4mo | 17.0 | Yes | No |
| 5 | No | Negative | < 10 | Yes/ ETV | 2yr, 11mo | 64.0 | No | No |

ADV, adefovir; anti-HCV, Hepatitis C antibody; anti-HDV, Hepatitis D antibody; FH, family history; HBeAg, Hepatitis B e-antigen; HBV, hepatitis B virus; HCC, hepatocellular carcinoma; HDV, Hepatitis D virus; IU/mL, international unit per millilitre; IVDU, intravenous drug use; LRC, liver-related complications; mo, month; MSM, men who have sex with men; No, number; Rx, medical therapy; VL, viral load; yr, year.

Supplement table 4 Comparison of laboratory findings of anti-HDV positive and anti-HDV negative patients

| **Laboratory findings** | **Anti-HDV negative** | | **Anti-HDV positive** | | **p-value** |
| --- | --- | --- | --- | --- | --- |
|  | **N** | **Number (%)/ Median (IQR)** | **N** | **Number (%)/ Median (IQR)** |  |
| Albumin (g/dL)  TB (mg/dL)  AST (U/L  ALT (U/L)  Platelet (x10^3^/µL)  AFP (ng/mL) | 678  682  690  690  670  642 | 4.30 (4.10-4.50)  0.68 (0.51- 0.97)  24.00 (20.00-34.00)  24.00 (18.00-36.00)  217.00 (173.50-261.50)  2.21 (1.51-3.61) | 4  4  4  4  3  4 | 4.20 (3.38-4.58)  0.99 (0.50-1.16)  44.50 (23.50-58.75)  56.00 (24.75-82.75)  209.00 (41.00-389.00)  1.56 (1.43-2.41) | 0.736  0.449  0.180  0.113  0.919  0.273 |

AFP, alpha-fetoprotein; anti-HDV, Hepatitis D antibody; ALT, alanine aminotransferase; AST, aspartate aminotransferase; mg/dL, milligram per decilitre; ng/mL, nanogram per millilitre; TB, total bilirubin; U/L, units per litre; µL, microlitre; IQR, interquartile range

Supplement table 5 Studies of prevalence of HDV infection in Thailand

| **Study** | **Year** | **Population** | **Test** | **Anti-HDV prevalence**  **N (%)** | **Detectable HDV RNA**  **N (%)** | **HDV genotype** |
| --- | --- | --- | --- | --- | --- | --- |
| Chainuvathi et al. | 1987 | Patients with HBV infection | N/A | 0.5% | N/A | N/A |
| Louisirirotchanakul et al. | 1988 | - IVDU with HBV infection  - Patients with HBV infection in the hospital setting | ELISA using competitive sandwich inhibition (Organon Teknika., Belgium) | 55/84 (65.5%)  3/135 (2.2%) | N/A  N/A | N/A  N/A |
| Theamboonlers  et al. | 2002 | - IVDU with HBV infection  - Asymptomatic HBV infection (blood donor) | EIA using ETI-MAK-3 and ETI-AB-DELTAK-2 (Dia-Sorin, Vercelli, Italy) | 12/55 (21.8%)  0/36 (0%) | 8/12 (66.6%)  - | 1  - |
| Jutavijittum et al. | 2002 | Asymptomatic HBV infection (blood donor) | EIA using ETI-AB-DELTAK-2 (Dia-Sorin, Vercelli, Italy) | 0/395 (0%) | - | - |
| Louisirirotchanakul et al. | 2002 | Hmong people with HBV infection in Northern Thailand | EIA (UBI, USA) | 1/150 (0.7%) | N/A | N/A |
| Hongjaisee et al. | 2020 | Migrant sex workers (Thaiyai, Burmese, Laos) in Chiangmai | EIA using DIA.PRO; Diagnostic Bioprobes, Milan, Italy  (Sn 98%, Sp 98%) | 0/45 (0%) | N/A | N/A |
| Ananchuensook  et al. (Our study) | 2023 | Patients with HBV infection in the hepatology clinics | EIA using DIA.PRO, Italy (Sn >98%, Sp >98%) and CLIA using LIAISON® XL MUREX Anti-HDV (DiaSorin Biotechnology, Italy) (Sn 99.5%, Sp 98.3-99.9%) | 4/702 (0.6%) | 2/4 (50.0%) | 1 |

anti-HDV, hepatitis D antibody; CLIA, chemiluminescence immunoassay; EIA, Enzyme link immunoassay; HBV, hepatitis B virus; Hepatitis D virus; IVDU, intravenous drug use; N/A, not applicable; Sn, sensitivity; Sp, specificity.
